# Johnston’s Fluid Beef

**Published:** 1882-04-20

**Authors:** 


JOHNSTON'S FLUID BEEF.—Robert Shoemaker & Co., Philadelphia, General Agents for United States. See the new advertisement of this article. It is truly an excellent preparation. We have tried it.

